# Sensory Attenuation of Auditory P2 Responses is Modulated by the Sense of Action Timing Control

**DOI:** 10.1111/psyp.70134

**Published:** 2025-09-03

**Authors:** Nathan Thomas Han, Tingting Yan, Ran Zhuang, Athanasios Vasileios Kokkinakis, Liyu Cao

**Affiliations:** ^1^ Department of Psychology and Behavioural Sciences Zhejiang University Hangzhou China; ^2^ The State Key Lab of Brain‐Machine Intelligence Zhejiang University Hangzhou China

## Abstract

Sensory attenuation is a well‐established phenomenon in which the neurophysiological response elicited by self‐initiated stimuli is attenuated compared to identical externally generated stimuli. This phenomenon is mostly studied by comparing the N1 and P2 components of the auditory ERP. Sensory attenuation has also been linked to our sense of agency and control. In the present study, we investigated the role of action timing control in sensory attenuation. Previous studies that investigated the attenuation of the N1/P2 components instructed participants to generate self‐initiated stimuli by having the participants perform a series of keypresses while EEG is recorded. ERP responses are then compared to a second condition where participants passively listen to identical sounds. Studies using this paradigm, known as the self‐stimulation paradigm, have used a wide range of stimulus onset asynchronies (SOAs) for keypress timing. However, the choice of SOA is rarely explained, perhaps due to an assumption of trial independence. We found that as SOA increased, participants enacted more action timing control to maintain the specified SOA level. The degree of P2 suppression also increased as participants enacted more control. Contrary to most studies in the literature, we did not find N1 suppression but instead found N1 enhancement. The results suggest that P2 suppression may be related to action timing control while N1 enhancement may reflect factors other than motor predictions, in line with more recent interpretations of the N1 suppression effect.

## Abstract 2

When we actively press a button to generate a sound, our neural response to the sound is attenuated relative to the same sound heard passively. This phenomenon, known as sensory attenuation, is generally studied by having participants perform strings of keypresses to generate sounds. Our study shows that the degree of attenuation depends on the length of time between keypresses. Participants must enact more action timing control as the length of time between keypresses increases. Our study also highlights the need to think of behavior beyond the level of the trial.

## 1 Introduction

Stimuli generated by person's own willed motor actions generally elicit a smaller electrophysiological response compared to physically identical stimuli that are externally generated (Cao, Thut et al. 2017; Horváth 2015; Hughes et al. 2013; Schafer and Marcus 1973). This phenomenon, known as sensory attenuation, has been widely and consistently reported in human studies measuring electromagnetic neural responses (Cao, Thut et al. 2017; Klaffehn et al. 2019; Mifsud et al. 2016; Paraskevoudi and SanMiguel 2023; Pinheiro et al. 2019; Seidel et al. 2023). This effect has most commonly been observed with the N1 (Bäß et al. 2008; Elijah et al. 2018; Jack et al. 2021; van Elk et al. 2014) and P2 components (Han et al. 2022; Horváth and Burgyán 2013) of the ERP (event‐related potentials).

Sensory attenuation has been attributed to forward models in which the brain uses a copy of the outgoing motor command to make predictions about the expected sensory consequences of self‐initiated movements (Korka et al. 2022; Miall and Wolpert 1996). Sensory predictions and sensory feedback are compared, with successfully predicted sensations being suppressed to prioritize the processing of unanticipated changes in our environment. Sensory attenuation has also been linked to our sense of agency and control (Han et al. 2021; Harrison et al. 2021; Hughes et al. 2013; Seidel et al. 2021). It has been argued that our phenomenological sense of agency is contingent upon successful suppression of self‐generated sensations. The characteristic abnormalities in sense of agency experienced by schizophrenia patients, for example, have been argued to reflect deficits in comparing self‐generated and externally generated stimuli (Fletcher and Frith 2009; Whitford 2019). However, it should be noted that though the idea of our phenomenological sense of agency being contingent upon successful suppression of self‐generated sensations is intuitive, there is yet to be any direct evidence that links the two phenomena together.

In studies that have investigated sensory attenuation through N1/P2 suppression (also known as the self‐stimulation paradigm (Han et al. 2021; Schafer and Marcus 1973)), control has mostly been investigated by manipulating the participant's belief or causality in their control of action effects (Gentsch and Schütz‐Bosbach 2011; Haggard 2017; Han et al. 2022; Seidel et al. 2021; Timm et al. 2016). For example, participants may be led to believe that their action had not caused the onset of a sound, despite actually having control over its onset (Timm et al. 2016), or that their control is limited as sounds only follow actions a certain percentage of the time (Han et al. 2022). However, this conception of control does not capture the sense of implicit action control that participants enact when performing the self‐stimulation task itself.

Experiments using the self‐stimulation paradigm typically include three conditions: the *motor‐auditory* (MA), *motor‐only* (MO), and *auditory‐only* conditions. Many, if not all, of the MA and MO conditions require participants to repeatedly perform a keypress at varying stimulus onset asynchronies (SOAs) ranging from 0.8 s/keypress to upward of > 5 s/keypress (Horváth 2015). The choice of the SOAs is rarely explained and, with a few exceptions (Horváth 2015; SanMiguel et al. 2013), is mainly thought of as a negligible factor in the self‐stimulation paradigm. This is perhaps because we tend to think of behavior as operating on the level of the trial (Gilden 2001; Huk et al. 2018) and for observed output variables to be the result of discrete mental states. The timing of keypresses for participants, however, is not trivial as participants presumably have to exercise more control to maintain keypress intervals of longer durations. Therefore, choosing when to make a keypress may be seen as a kind of implicit sense of action control. Here, the exercise of control likely involves a range of processes including working memory and duration estimation.

Fractal analyses, such as the detrended fluctuation analysis (DFA), is one method by which to measure the level of implicit action control exercised by participants. It is used to characterize the autocorrelation of a time series and yield a measure known as the *α* exponent. In the psychological literature, the *α* exponent, a number ranging from 0 to 2, has been argued to be a measure of the exercise of control over event parameters such as timing (Dingwell and Cusumano 2010; Diniz et al. 2011; Likens et al. 2015). When one attempts to enact control over an event parameter (e.g., a keypress every *X* seconds or maintaining a particular walking speed), statistical properties of *persistence* (when the *α* exponent is between 0.5 and 1^1^) or *anti‐persistence* (when the exponent is between 0 and 0.5) tend to emerge in the behavior time series (Dingwell and Cusumano 2010). An intuitive way of thinking about persistence in keypress behavior is that, on average, long keypress intervals will be followed by long intervals and short intervals are more likely to be followed by short intervals. On the other hand, anti‐persistence means that long keypress intervals will more likely be followed by short intervals and vice versa. For example, Likens et al. (2015) conducted a study wherein participants either steered a wheel around a curve, thus requiring control at a constant angle, or along a straight track. Their results showed persistent behavior to be higher for steering behavior on the curved track compared to the straight track. Such methods have also been applied to time series of a range of behavioral phenomena including walking (Dingwell and Cusumano 2010; Hausdorff et al. 1997) and continuous tapping behavior, with several studies finding more fractal behavior^2^ as the time between taps increased (Madison 2004; Madison and Delignières 2009)‐ perhaps reflecting the greater degree of implicit control needed as the timing requirements increased. Furthermore, rhythmic motor activity, such as finger tapping, has been shown to be involved in guiding fluctuations of attention toward temporally regular events (Morillon et al. 2014, 2015) and such an interaction may also be at play in the self‐stimulation task. For example, Morillon et al. (2014) found that overt rhythmic finger movements helped to enhance attention toward target tones amidst a stream of distractor tones, demonstrating that rhythmic motor activity can guide attention at important points in time to facilitate sensory processing. Different temporal intervals between sounds, specified by different SOA levels, may also affect the allocation of attention during the self‐stimulation task. Given that the N1 and P2 amplitudes have been shown to be affected by attention (Crowley and Colrain 2004), the interaction between temporal attention and motor activity may therefore have an effect on component amplitudes.

In the current study, we used SOA levels of keypresses every 0.8, 1.6, and 3.2 s, which were based on those used by SanMiguel et al. (2013). Participants took part in three experimental sessions where they completed the MA, MO, and *auditory–visual* (AV, replacing the *auditory‐only* condition of past studies; see *Stimuli, Materials, and Procedure* for further details) conditions of the self‐stimulation experiment. We also recorded the keypress timings for the MA and MO conditions to compute *α* exponents across the three SOA levels. We were interested in studying how the enactment of control—indexed by the *α* exponent—would be related to the degree of sensory attenuation. In this sense, the degree of sensory attenuation served as a neural correlate for this implicit sense of action control. Like most self‐stimulation experiments, we looked at the N1 and P2 components of the ERP. We hypothesized that as participants exerted more control over their keypressing behavior, the degree of attenuation in the P2 component would also increase, as P2 suppression has been linked to participant control over action effects (Timm et al. 2016; Seidel et al. 2021). Furthermore, some of the mental processes possibly involved with implicit action control, such as working memory and duration estimation (Duzcu et al. 2019; Lefebvre et al. 2005), have been associated with modulations of P2 magnitude. For example, Duzcu et al. (2019) found P2 amplitudes to increase when participants had to indicate whether one temporal interval—marked by two tones—was longer or shorter than another temporal interval, versus another task where they only had to indicate whether a temporal interval matched that of a trained, reference interval. Because the aforementioned effects tend to be associated more with the P2 component, we did not expect the degree of N1 attenuation to correlate with the implicit action control and instead hypothesized that the degree of attenuation would remain stable across SOA levels.

## 2 Method

### 2.1 Participants

The final sample for data analysis included 21 participants (12 female, mean age = 22 years, SD = 2), recruited from a local participant pool. Four participants were excluded from the initial 25 participants for reasons including an excessive number of bad trials after artifact detection (2; exclusion if more than 50% bad trials within a single condition), data that was corrupted and so could not be preprocessed (1), and failure to complete the experiment (1). All participants gave written informed consent before the experiment. All participants were debriefed and received monetary payment after the experiment. Using the statistical power calculator developed by Hall et al. (2023) based on Monte Carlo simulations of ERP data from a passive listening task, we estimated a statistical power between 0.87 and 0.97 for detecting differences in the N1 and P2 components, respectively. The values from the statistical power calculator were obtained via the parameters of 520 trials (rounded up from 512 trials after trial exclusion‐ described further in *EEG Recording and Analysis*), 20 participants (rounded down from 21), and a chosen effect magnitude of 1 μV. The chosen effect magnitude was taken from a range of articles including Han et al. (2022), Kaiser and Schütz‐Bosbach (2018), and Klaffehn et al. (2019). The experiment was conducted in accordance with the Declaration of Helsinki (2013) and was approved by the Ethics Committee of the Department of Psychology and Behavioral Sciences, Zhejiang University (*2023[049]*). Methods were not preregistered prior to conducting this study.

### 2.2 Stimuli, Materials, and Procedure

The audio stimulus was a sinusoid tone of 1000 Hz, 100 ms duration, with a 5 ms linear rise/fall time. Audio stimuli were sent to participants through Beyerdynamic DT 770 Pro headphones. Audio input/output was controlled by a specially written MATLAB script and was delivered via Psychtoolbox‐3 (Kleiner et al. 2007). Visual stimuli were displayed on a 24‐in. LCD monitor. Participants sat approximately 60 cm away from the monitor.

**FIGURE 1:**
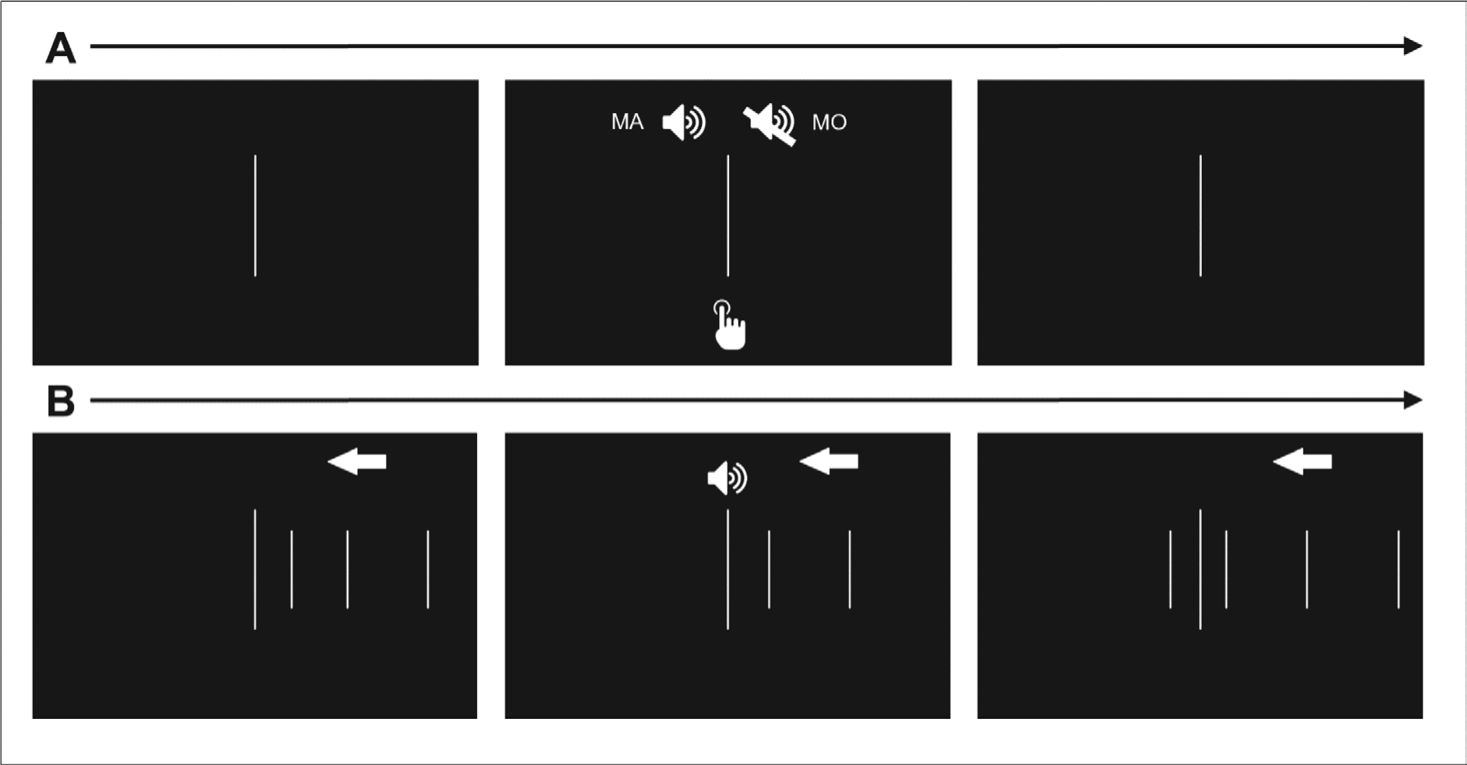
Schematic of experimental protocol. In the MA and MO conditions (*row A*), participants fixate on a white, central line and perform keypresses at SOAs of 0.8, 1.6, or 3.2 s per keypress (SOAs are divided by blocks). In the MA condition, the keypress is followed by a sound 100 ms later; keypresses in the MO condition are not followed by a sound. In the AV condition (*row B*), participants again fixate on a white, central line. Shorter white lines appear from the right side of the screen and move toward the central line at a rate of 1.67 cm per second. When the moving line crosses over the central line, participants hear a sound; the moving line then continues moving until it reaches the left side of the screen. The spacing of the moving lines is set so that the timing of the sounds match that of the keypress intervals in the participants MA condition attempt.

The visual stimuli were adapted from the experimental paradigm used by Whitford et al. (Whitford et al. 2017). During the experiment, participants fixated on a white, central fixation line of approximately 10 cm in length (see Figure 1). In the MA condition, participants fixated on the fixation line and performed keypresses that were followed by an auditory tone 100 ms later. Participants pressed the “k” key with their right index finger once every 0.8 s, 1.6 s, or 3.2 s for 525 keypresses per SOA level. Each string of 525 keypresses was performed within a single, separate block. The long string of keypresses was necessary in order to use the DFA procedure, which requires a time series of at least ~500 values. For the MA condition, the time taken between each keypress was recorded for analysis and used for sound onset times for the later AV condition.

The MO condition was nearly identical to the MA condition except that no sound followed the keypress. For both the MA and MO conditions, participants did not receive feedback on their keypress intervals during the block as the keypress time series needed to be uninterrupted.

In the AV condition, participants simply fixated on the central fixation line and listened to sounds without performing any keypresses. From the right end of the screen, white vertical lines of approximately 5.5 cm in length appeared and traveled toward the left end of the monitor at a rate of approximately 1.67 cm per second (see Figure 1). When the moving white line crossed the central fixation line (taking approximately 16 s for the line to move from the right side of the screen to the central fixation line), the auditory tone would be played. The white lines appearing from the right were spaced apart in a manner so that the timing of sound onset would match the timing of the time series attained by the participants MA condition keypress timings. In this manner, the timing of sound onset would be consistent across the MA and AV conditions.

Prior to the actual testing blocks, participants went through a practice block. They completed practice blocks for the MA and MO conditions. However, because participants were not required to perform any keypresses, there was no practice session for the AV condition. During the practice run, participants listened to tones at the rates of the specified SOA levels for 30 s and were asked to replicate the timing of the tones through keypresses. They performed 30 keypresses for the 0.8 SOA level, 20 keypresses for the 1.6 level, and 10 keypresses for the 3.2 level. The mean keypress timing was calculated for the practice block, and if it was outside ±1.3 times the required SOA rate, they were asked to repeat the practice block. For the MA condition, their keypresses were followed by an identical sound to the actual MA testing block sound. For the MO condition, their keypresses were not followed by a sound.

In total, each participant completed nine experimental blocks, corresponding to the MA, MO, and AV conditions for each SOA level of 0.8, 1.6, and 3.2 s per keypress/sound. Each block contained 525 samples of keypresses/sounds. The time taken for the 0.8 SOA level was less than the 1.6 SOA level, and the 1.6 SOA level was less than the 3.2 SOA level. On average, the 0.8 SOA level took 7.02 min, the 1.6 SOA level took 13.8 min, and the 3.2 SOA level took 27.32 min. Participants could take self‐timed breaks after each block. Due to the length of the experiment, participants completed three two‐hour sessions. Each session occurred on consecutive days or with a gap of one day between the sessions. Sessions were also run at the same time of the day as the previous session/s. The order of conditions and SOA levels was counterbalanced, but the AV condition only happened after a MA condition had already occurred, as the timing of sound onset in the AV condition was based on the keypress timing of the MA condition. Each session only ran one condition but contained all three SOA levels. For example, a participant might complete the MO condition (with all three SOAs) during session one, the MA condition during session two, and finally the AV condition during session three.

During behavioral testing, EEG was recorded with a BioSemi ActiveTwo system from 64 Ag/AgCl active electrodes (P1, FPz, FP2, AF7, AF3, AFz, AF4, AF8, F7, F5, F3, F1, Fz, F2, F4, F6, F8, FT7, FC5, FC3, FC1, FCz, FC2, FC4, FC6, FT8, T7, C5, C3, C1, Cz, C2, C4, C6, T8, TP7, CP5, CP3, CP1, CPz, CP2, CP4, CP6, TP8, P9, P7, P5, P3, P1, Pz, P2, P4, P6, P8, P10, PO7, PO3, POz, PO4, PO8, O1, Oz, O2, Iz). A vertical EOG was recorded by placing an electrode above and below the left eye; a horizontal EOG was recorded by placing an electrode on the outer canthus of each eye. Electrodes were also placed on each mastoid and the nose. During data acquisition, the common mode sense (CMS) and driven right leg (DRL) electrode sites served as the reference, and the sampling rate was 1024 Hz. Impedances were kept below 20 kΩ.

### 2.3 EEG Analysis

Offline analysis was run using EEGLAB version 2024.2.1 (Delorme and Makeig 2004) and ERPLAB version 12.00 (Lopez‐Calderon and Luck 2014) MATLAB packages. We re‐referenced the EEG data offline to the mastoid electrodes, as is common in studies investigating the components of interest (N1 and P2) for sensory attenuation. Data were band‐pass filtered from 0.1 to 30 Hz using a non‐causal Butterworth filter (slope = 12 dB/octave) and then notch‐filtered (50 Hz) to remove mains artifacts. The filtered data were segmented into 600 msec epochs, from 200 msec before the sound onset to 400 msec after the sound onset (with sound onset at time 0). Epochs were baseline‐corrected to the mean voltage from −200 to 0 msec.

Ocular and artifact correction was performed using independent component analysis (ICA) after epoching and on each condition separately (the MA, MO, and the AV conditions). Components for eye movement artifacts as well as bad channels and muscle artifacts were identified using the ICLabel classifier built into EEGLab (using an initial artifact identification threshold of 80%) and cross‐referenced using the guide from Chaumon et al. (2015). We excluded all epochs with signals exceeding peak‐to‐peak amplitudes of 200 μV. We analyzed the amplitudes of the N1 and P2 components of the auditory‐evoked potential, which were calculated as the average voltage within time windows (30 msec width), the centers of which were defined using the collapsed localizer approach (Luck and Gaspelin 2017). The collapsed localizer approach is a technique whereby one first averages (or collapses) the ERP waveforms across all conditions for all participants. The components of interest (e.g., N1 and P2) are identified on this collapsed waveform, and a time window is centered around these peaks, which is then used for the statistical analysis of the original (or uncollapsed) waveforms (Luck and Gaspelin 2017). The collapsed localizer was applied after the first 13 epochs were removed to match the removal of the first 13 samples in the time series for the DFA analysis (more on this in *Keypress Timing Analysis*). Electrodes of interest were pooled across Fz, FCz, and Cz for the N1 component and FCz, Cz, and CPz for the P2 component. Electrodes were chosen to be consistent with Whitford et al. (2017) and Han et al. (2022) and referenced with topographies to check if electrode choices were appropriate. Epochs from the MO condition were subtracted from those of the MA condition to create a motor‐controlled (C‐MA) ERP.

There was an average of 506.00 (SD = 5.50) usable epochs in the MA‐0.8 condition, 504.14 (SD = 8.51) in the MA‐1.6 condition, and 505.33 (SD = 4.42) in the MA‐3.2 condition. There was an average of 505.95 (SD = 5.34) usable epochs in the MO‐0.8 condition, 501.86 (SD = 863) in the MO‐1.6 condition, and 503.19 (SD = 9.55) in the MO‐3.2 condition. Finally, there was an average of 506 (SD = 6.22) usable epochs in the AV‐0.8 condition, 506.19 (SD = 6.54) in the AV‐1.6 condition, and 504.81 (SD = 6.62) in the AV‐3.2 condition. Trial numbers were submitted to a 3 (Production: MA, MO, AV) × 3 (SOA: 0.8, 1.6, 3.2) repeated measures ANOVA, showing no significant differences among trial numbers for *Production* (*F*(2, 40) = 1.66, *p* = 0.203, $\eta_{p}^{2}$ = 0.08) or *SOA* (*F*(2, 40) = 1.27, *p* = 0.292, $\eta_{p}^{2}$ = 0.06).

### 2.4 Keypress Timing Analysis

Timing fluctuations of the keypresses (i.e., the inter‐keypress intervals; IKIs) were analyzed using detrended fluctuation analysis (DFA) (Peng et al. 1994) via the Python package *Antropy* version 0.1.6. The DFA procedure was done for each SOA level of both MA and MO conditions, yielding six values per participant. In each SOA level, the 525 keypresses yielded 524 IKIs. We discarded the first 12 IKIs to remove possible transients in keypress timing, leaving 512 IKIs (or samples) in the time series for analysis. The DFA algorithm consists of five steps (see Arsac and Deschodt‐Arsac (2018) and Likens and Stergiou (2020) for more detailed explanations) and is widely used for its versatility:

1. Subtract the mean of the time series from each sample (i.e., each value in the time series) to create a centered time series. Integrate the centered time series by cumulatively summing each element of the time series to create an integrated time series. That is, in the integrated time series, the sample *n* would be the sum of the first *n* samples from the centered time series. For example, sample 10 of the integrated time series would be the sum of the first 10 samples of the centered time series.
2. Divide the integrated time series into a sequence of usually nonoverlapping windows, with each window containing *n* samples. This step, as well as steps three and four, will be repeated for windows of different sizes of *n*. Typically, *n* ranges from a minimum value such as 4 and can increase up to *n* = *N*/2, *N* being the size of the original time series. For the time series of *N* = 512, the *Antropy* package used a base value of 4 and successively multiplied by a factor of 1.2 to achieve window sizes of 4, 5, 6, 8, 9, 11, 14, 17, 20, 24, 29, 35, and 42, which were based on Hardstone et al.'s (2012) recommendation to keep *n* no smaller than 4 and no larger than 10% of the time series size.
3. Fit a linear regression line over the samples in the window and subtract the fitted line from the data in each window. This is the *detrending* part of the analysis.
4. The root‐mean‐squared (RMS) residual is calculated for each window of size *n*. This is done by taking the detrended window in step three and then squaring each value (i.e., the residual) of the detrended window, calculating the mean of the squared residuals, and then taking the square root of the mean of the squared residuals. This yields an RMS residual and essentially characterizes the variability of the detrended fluctuations within the window. The RMS residuals of each window size *n* are averaged together.
5. Steps two to four are repeated for all the sizes of *n*. Plot log(*F*(*n*)) against log(*n*), with *n* being the window size and *F*(*n*) being the averaged RMS residuals of window size *n*. Finally, a linear regression line is fitted on the log–log plot. The slope of the line corresponds to the exponent *α*, which we discuss below.

Analyses such as the DFA try to capture the fractal qualities of time series. What fundamentally characterizes these fractal qualities is that at different scales of observation (i.e., the different window sizes *n*), one should observe similar statistical properties between windows such as variability or correlation structure (i.e., the RMS residual for windows of size *n*). All fractal analyses including the DFA yield the fractal dimension by using the slope of the regression line fitted on a log–log plot of the *scale* (i.e., window size) and *measured property* (i.e., RMS residuals). The *α* exponent provides an indicator of the statistical persistence (or positive autocorrelation) or anti‐persistence (or negative autocorrelation) of a time series. When the *α* exponent sits between 0 and 0.5, the time series is said to be anti‐persistent. An *α* exponent between 0.5 and 1 reflects a persistent time series. On the other hand, an *α* exponent > 1 may indicate that the time series is nonstationary, which may be addressed with further preprocessing of the initial time series. However, due to potential biases of the DFA procedure, several studies have advised that time series should only be classified as nonstationary if they exceed the value of 1.2 (Arsac and Deschodt‐Arsac 2018; Delignières and Marmelat 2012; Ihlen 2012). Our highest observed *α* exponent was 1.19, with a total of six *α* exponents > 1. For more detailed information regarding the *α* exponent and DFA analysis, please refer to the Supporting Information.

### 2.5 Statistical Analyses

Statistical analyses were performed using *JASP* version 0.19.1 (JASP Team 2024). Non‐sphericity was corrected using the Greenhouse–Geisser method (Geisser and Greenhouse 1958) when identified via Mauchly's test (Mauchly 1940).

For behavioral results, main effects for IKIs and *α* exponents were submitted to a 2 (Production: MA, MO) × 3 (SOA: 0.8, 1.6, 3.2) repeated measures ANOVA. For the N1 and P2 components, mean voltage in the analysis window was submitted to a 2 (Production: C‐MA, AV) × 3 (SOA: 0.8, 1.6, 3.2) repeated measures ANOVA. A series of paired‐samples *t*‐tests were conducted to evaluate differences in interaction effects and other contrasts of interest. The Benjamini–Hochberg method (Benjamini and Hochberg 1995) was used to adjust *p* values (*p*_adj_) and control the false discovery rate.

A linear mixed effects model analysis was conducted to assess the relationship between the implicit sense of action control and its effect on sensory processing. The linear mixed effects model was performed with the *jamovi* software version 2.3.28.0 (2024). No transformations were made to the data for the purposes of meeting assumptions. For both N1 and P2 analyses, there were 63 observations and 21 groups (for 21 participants and 3 observations (3 *α* exponents) per participant) in each condition.

For the N1 component, the model (*N1‐ C‐MA ~ 1 + α‐ MA + N1‐ AV + MA‐ IKI +* (*1 | Participant*)) consisted of the N1 amplitude of the corrected MA condition as the output variable, fixed effects of the *α* exponent in the MA condition, MA condition IKI, and N1 amplitude in the AV condition, and random intercepts for Participants. For the P2 component, the model (*P2‐ C‐MA ~ 1 + α‐ MA + P2‐ AV + MA‐ IKI +* (*1 | Participant*)) consisted of the P2 amplitude of the corrected MA condition as the output variable, fixed effects of the *α* exponent in the MA condition, MA condition IKI, and P2 amplitude in the AV condition, and random intercepts for Participants. Random slopes for effects of *α ‐MA* and *N1‐ AV/P2‐ AV* were not included in the analysis as slopes were highly correlated, leading to nearly identical slopes for most participants and an (almost) singular fit. Therefore, for the final model, only the random intercept (*Participant*) was included.

## 3 Results

### 3.1 Behavior Results

Participants could maintain the IKI as instructed. When actions were followed by sound feedback, the average IKI was 0.85 s (SD = 0.16) for the MA‐0.8 condition, 1.71 s (SD = 0.28) for the MA‐1.6 condition, and 3.18 s (SD = 0.48) for the MA‐3.2 condition. When actions were not followed by sound feedback, the average IKI was 0.75 s (SD = 0.14) for the MO‐0.8 condition, 1.45 s (SD = 0.25) for the MO‐1.6 condition, and 3.06 s (SD = 0.67) for the MO‐3.2 condition. The keypress timing was submitted to a 2 (Production: MA, MO) × 3 (SOA: 0.8, 1.6, 3.2) repeated measures ANOVA. Significant main effects of *Production* (*F*(1, 20) = 7.15, *p* = 0.015, $\eta_{p}^{2}$ = 0.26; with a longer IKI in the MA condition compare to the MO condition) and *SOA* (*F*(1.18, 23.57) = 287.80, *p* < 0.001, $\eta_{p}^{2}$ = 0.94; the IKI increased from SOA 0.8 to SOA 3.2) were found. The interaction effect *Production × SOA* was not significant (*F*(1.21, 24.27) = 0.86, *p* = 0.384, $\eta_{p}^{2}$ = 0.04). Non‐sphericity was identified in the *SOA* and *Production × SOA* effects using Mauchly's test (Mauchly 1940) and corrected using the Greenhouse–Geisser method (Geisser and Greenhouse 1958).

**FIGURE 2:**
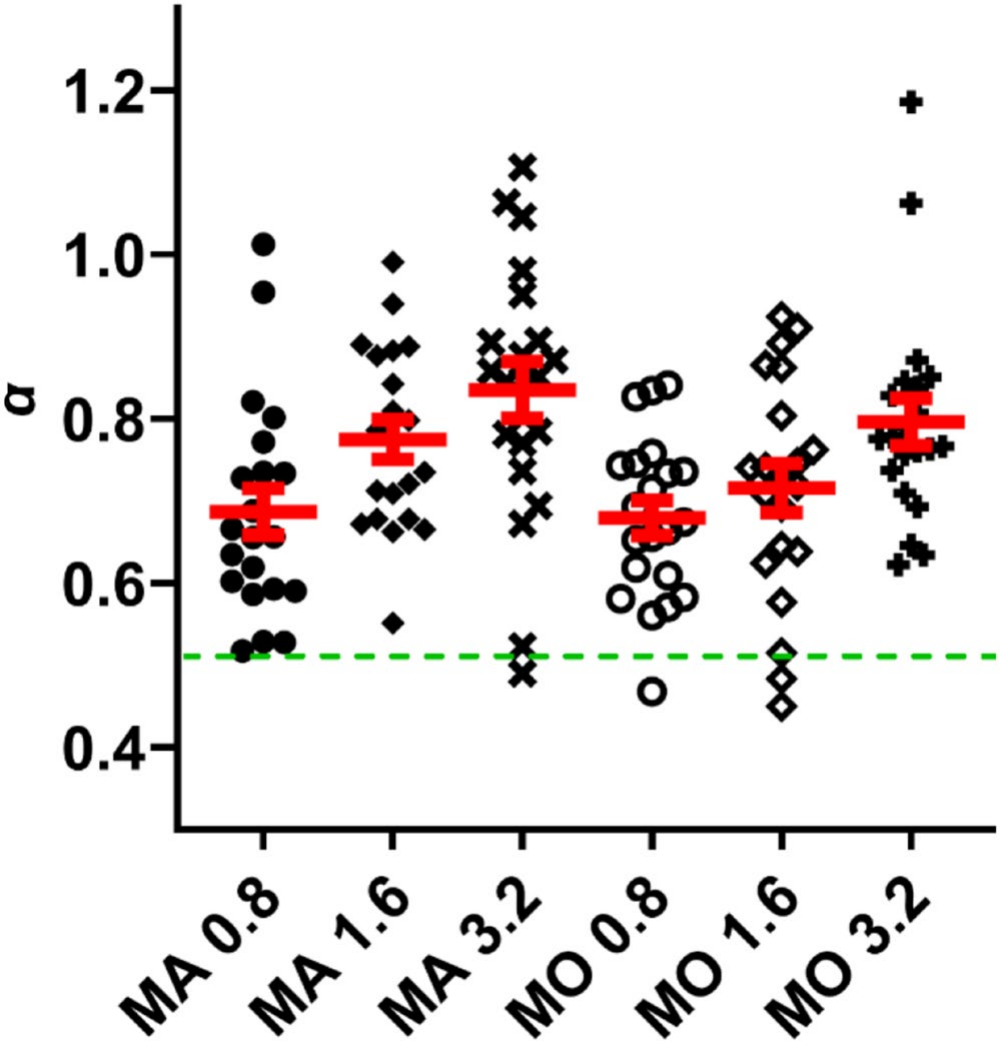
The *α* exponent values across MA/MO and SOA conditions. The middle red line represents the mean and the error bar represents the standard error. The green dashed line represents the 0.5 level for the *α* exponent.

Regarding the *α* exponents (Figure 2), a 2 (Production: MA, MO) × 3 (SOA: 0.8, 1.6, 3.2) repeated measures ANOVA yielded a significant main effect of *Production* (*F*(1, 20) = 5.12, *p* = 0.035, $\eta_{p}^{2}$ = 0.20). The MA condition (mean = 0.77, SD = 0.15) had larger *α* exponents than the MO condition (mean = 0.73, SD = 0.13). The main effect of *SOA* was also significant (*F*(2, 40) = 11.04, *p* < 0.001, $\eta_{p}^{2}$ = 0.36). The SOA‐3.2 condition (mean = 0.82, SD = 0.14) had larger *α* exponents than the SOA‐1.6 condition (mean = 0.75, SD = 0.13; *t*(20) = 2.64, *p* = 0.016, *d* = 0.58; *p*_adj_ = 0.024) and the SOA‐0.8 condition (mean = 0.68, SD = 0.11; *t*(20) = 4.25, *p* < 0.001, *d* = 0.93; *p*_adj_ = 0.003). The SOA‐1.6 condition also had larger *α* exponents than the SOA‐0.8 condition (*t*(20) = 2.33, *p* = 0.030, *d* = 0.51; *p*_adj_ = 0.030). The *Production × SOA* interaction was not statistically significant (*F*(2, 40) = 0.66, *p* = 0.522, $\eta_{p}^{2}$ = 0.03).

The DFA procedure is known to be affected by the length of the time series, with shorter series lengths potentially producing more biased *α* exponent estimates (Eke et al. 2002). Furthermore, it has been argued that fractal properties may arise due to the distribution of values exceeding a critical boundary in the time series (Werner 2010), which may potentially implicate the larger *α* exponents in the MA condition compared to the MO condition and increased *α* exponents with increased SOA levels as the SD of IKIs was larger in the MA condition compared to the MO condition, and as SOA levels increased. To exclude these possibilities, we randomly shuffled all time series within each SOA and production condition, a measure used by Hausdorff et al. (1996) and Madison (2004).

By shuffling the time series, the mean and SD of the IKIs remained the same, but the sequential order (the basis of the DFA analysis) would be different. We performed this procedure 500 times and ran repeated measures ANOVAs for each loop, yielding 500 *F* values for the main effect of SOA and 500 *F* values for the main effect of Production. The *F* value for the main effect of SOA with the original data was smaller than only one of the shuffled F values (equivalent to *p* = 0.002). Similarly, the *F* value for the main effect of Production with the original data was smaller than 19 of the shuffled *F* values (equivalent to *p* = 0.038). Thus, the main effects of SOA and Production on *α* exponents were likely not artifacts of different IKIs.

### 3.2 EEG Results

#### 3.2.1 N1

**FIGURE 3:**
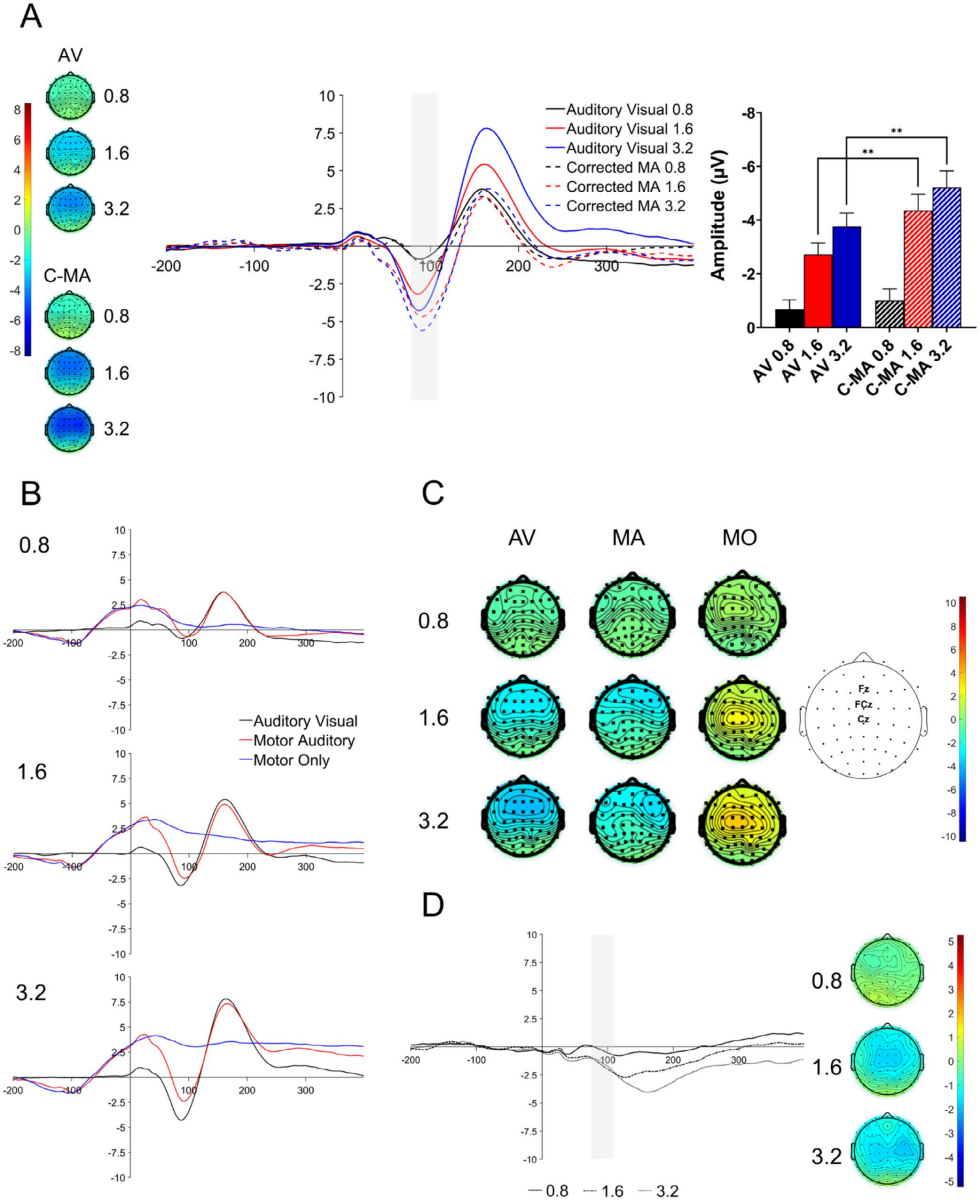
Analyses of the N1 component. The N1 component was measured at electrodes Fz, FCz, and Cz, with time window 76.8–106.8 ms (A) Waveforms showing the AV and C‐MA ERPs as well as corresponding topographies across all SOA levels. Time window of analysis is shown via the light gray bar. Bar plots illustrating N1 amplitudes across conditions and SOA levels and significant contrasts. Error bars show SEM. Asterisks represent levels of significance (**: *p* < 0.01). (B) Grand average waveforms for the AV condition (black lines), MA condition (red lines), and MO condition (blue lines) across SOA conditions (0.8, 1.6, 3.2). Sound onset was at time 0 ms for the MA and AV conditions. The keypress was made at −100 ms in MA and MO conditions. (C) N1 scalp topographies for the AV, MA, and MO conditions. Electrodes of interest and locations are shown to the right of the scalp topographies. (D) Difference waves and scalp topographies for the effects (C‐MA minus AV) at the N1 time window. For the ERP waveforms, the SOA levels are represented via the separate lines (0.8: Solid line; 1.6: Dashed/dotted lines; 3.2: Dotted lines). The keypress was performed at time point −100 ms during the [−200 0] ms baseline period. Sound onset was at time 0 ms.

Figure 3A shows the grand average N1 component elicited in the C‐MA (i.e., the corrected auditory ERP in the MA condition through subtracting the ERP in the MO condition) and AV conditions. The time window for the N1 analysis was 76.8–106.8 ms. The main effect of *SOA* was significant (*F*(2, 40) = 39.98, *p* < 0.001, $\eta_{p}^{2}$ = 0.67), as was the main effect of *Production* (*F*(1, 20) = 8.26, *p* = 0.009, $\eta_{p}^{2}$ = 0.29). There was also a significant *Production×SOA* interaction (*F*(2, 40) = 4.06, *p* = 0.025, $\eta_{p}^{2}$ = 0.17). These results show that the N1 amplitude of the C‐MA condition was enhanced relative to the AV condition, although not at all SOA levels.

We also conducted paired‐samples *t*‐tests comparing N1 amplitudes at each SOA level. The contrast between the AV‐0.8 and C‐MA‐0.8 was not significant (*t*(20) = 0.73, *p* = 0.473, *d* = 0.16; *p*_adj_ = 0.473). However, the contrast between AV‐1.6 and C‐MA‐1.6 was significant (*t*(20) = 2.90, *p* = 0.009, *d* = 0.63; *p*_adj_ = 0.014), as was the contrast between AV‐3.2 and C‐MA‐3.2 (*t*(20) = 3.13, *p* = 0.005, *d* = 0.68; *p*_adj_ = 0.014). The results here demonstrate that an N1 enhancement for the C‐MA condition relative to the AV condition occurred only for the 1.6 s and 3.2 s SOA levels.

Finally, we computed difference waves between the AV and C‐MA ERPs (Figure 3D) and conducted paired‐samples *t*‐tests to compare differences between levels of enhancement between the SOA levels. Compared to the 0.8 s SOA, both the 1.6 s SOA (*t*(20) = 2.38, *p* = 0.027, *d* = 0.52; *p*_adj_ = 0.041) and 3.2 s SOA conditions (*t*(20) = 2.41, *p* = 0.025, *d* = 0.53; *p*_adj_ = 0.041) were significantly different. Furthermore, there was no significant difference between the 1.6 s and 3.2 s SOAs (*t*(20) = −0.39, *p* = 0.701, *d* = −0.09; *p*_adj_ = 0.701).

The N1 is known to involve a combination of several subcomponents (Näätänen and Picton 1987) such as the sensory processing‐related Component 1 and the unspecific component (Component 3) that is mostly associated with motor activity instead of any sensory‐specific processing. It has been argued that the N1 attenuation effect mostly reflects suppression of the unspecific component (SanMiguel et al. 2013) due to between‐condition differences in the orientation behavior of participants toward sound onset (e.g., sounds are not temporally predictable in the traditional auditory‐only condition so participants orient toward sound onset). However, the unspecific component has a relatively long refractory period and so was only observed in the 3.2 SOA level in the study by SanMiguel et al. (2013). Differences in N1 amplitude across SOA levels may thus be a combination of different subcomponents, with these subcomponents contributing differently across SOA levels. The original ERPs (Figure 3B) showed relatively stable latencies for the N1 components of the MA and AV conditions as well as consistent topographies across SOA levels, most importantly the 3.2 SOA level (Figure 3C), suggesting that the difference in component amplitudes may be genuinely modulated by the differences in sensory‐specific processing across SOA levels as opposed to the unspecific component. This may be because the visual stimulation of the AV condition acted to limit orientation responses to sound onset as sounds were predictable. Furthermore, the effect difference waves (C‐MA minus AV, see Figure 3D) as well as the corresponding topographical map do not suggest any large contribution from an alternative ERP component as topographies share largely the same distribution as the underlying (original) ERPs.

#### 3.2.2 P2

**FIGURE 4:**
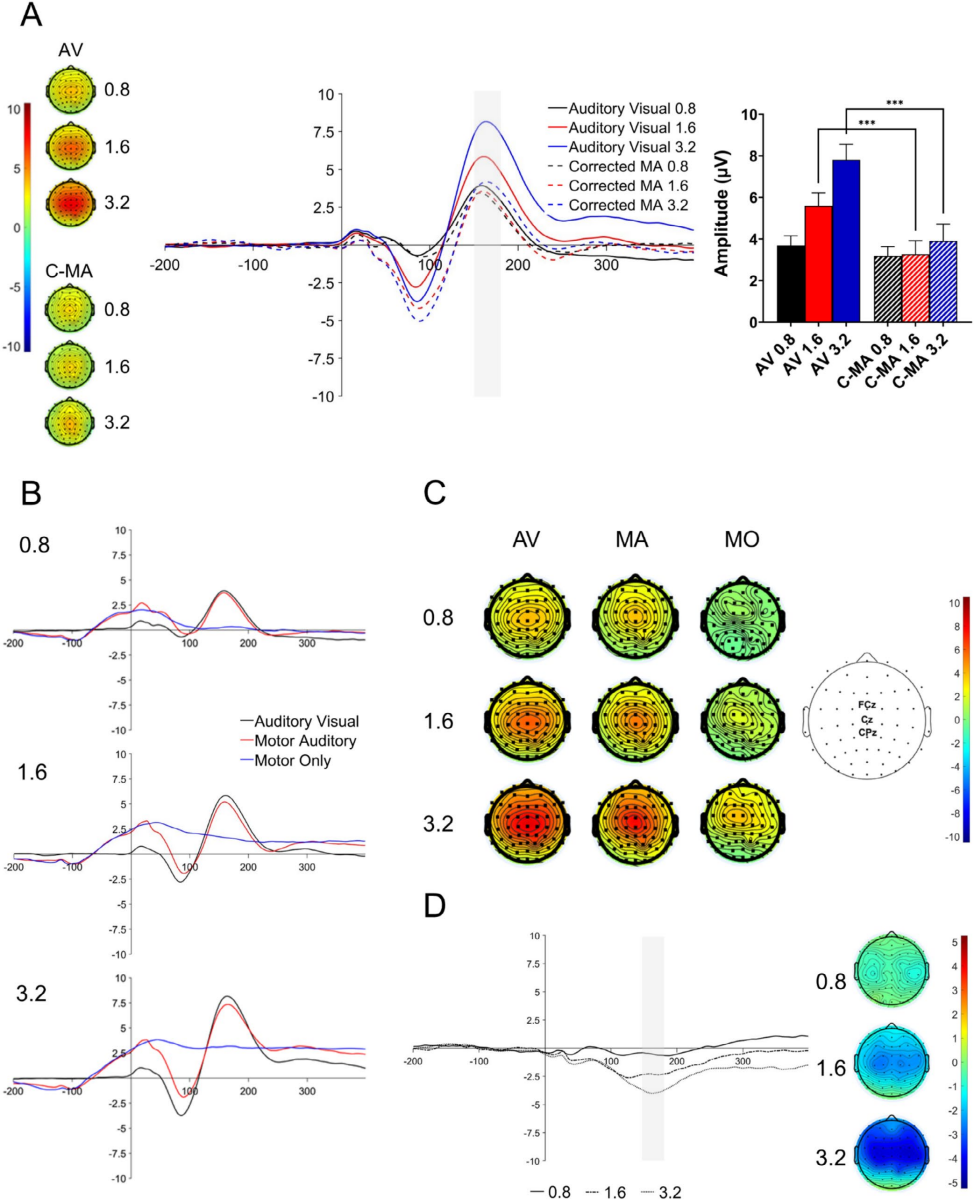
Analyses of the P2 component. The P2 component was measured at electrodes FCz, Cz, and CPz, with time window 146.13–176.13 ms (A) Waveforms showing the AV and C‐MA ERPs as well as corresponding topographies across all SOA levels. Time window of analysis is shown via the light gray bar. Bar plots illustrating P2 amplitudes across conditions and SOA levels and significant contrasts. Error bars show SEM. Asterisks represent levels of significance (**p < 0.01). (B) Grand average waveforms for the AV condition (black lines), MA condition (red lines), and MO condition (blue lines) across SOA conditions (0.8, 1.6, 3.2). Sound onset was at time 0 ms for the MA and AV conditions. The keypress was made at −100 ms in MA and MO conditions. (C) P2 scalp topographies for the AV, MA, and MO conditions. Electrodes of interest and locations are shown to the right of the scalp topographies. (D) Difference waves and scalp topographies for the effects (C‐MA minus AV) at the P2 time window. For the ERP waveforms, the SOA levels are represented via the separate lines (0.8: Solid line; 1.6: Dashed/dotted lines; 3.2: Dotted lines). The keypress was performed at time point −100 ms during the [−200 0] ms baseline period. Sound onset was at time 0 ms.

Figure 4A shows the grand average P2 component elicited in the C‐MA and AV conditions. The time window for the P2 analysis was 146.13–176.13 ms. The main effect of *SOA* was significant (*F*(1.48, 29.60) = 18.04, *p* < 0.001, $\eta_{p}^{2}$ = 0.47), as was the main effect of *Production* (*F*(1, 20) = 61.91, *p* < 0.001, $\eta_{p}^{2}$ = 0.76). There was also a significant *Production × SOA* interaction (*F*(1.95, 39) = 14.96, *p* < 0.001, $\eta_{p}^{2}$ = 0.43). These results show that the P2 amplitude of the C‐MA condition was attenuated relative to the AV condition depending on SOA levels.

We conducted paired‐samples *t*‐tests comparing P2 amplitudes for production conditions (C‐MA and AV conditions) at each SOA level. The contrast between the AV‐0.8 and C‐MA‐0.8 was not significant (*t*(20) = 1.62, *p* = 0.121, *d* = 0.35; *p*_adj_ = 0.121). However, the contrast between AV‐1.6 and C‐MA‐1.6 was significant (*t*(20) = 4.77, *p* < 0.001, *d* = 1.04; *p*_adj_ = 0.002), as was the contrast between AV‐3.2 and C‐MA‐3.2 (*t*(20) = 7.24, *p* < 0.001, *d* = 1.58; *p*_adj_ = 0.002). The results here demonstrate that P2 attenuation for the C‐MA condition relative to the AV condition occurred only for the 1.6 s and 3.2 s SOA levels.

The original ERPs (Figure 4B) and scalp topographies (Figure 4C) for the P2 component show the P2 component waveform and scalp distribution to be highly consistent across SOA levels. Effect difference waves and topographic distribution (Figure 4D) are also consistent with the underlying ERP components and scalp topographies.

Finally, we again computed difference waves between the AV and C‐MA ERPs (Figure 4D) and conducted paired‐samples *t*‐tests to compare differences between levels of attenuation between the SOA levels. There was a significant difference between the 0.8 s and 1.6 s SOAs (*t*(20) = 3.08, *p* = 0.006, *d* = 0.67; *p*_adj_ = 0.009), as well as between the 0.8 s and 3.2 s SOAs (*t*(20) = 5.08, *p* < 0.001, *d* = 1.11; *p*_adj_ = 0.003). There was also a significant difference between the 1.6 s and 3.2 s SOAs (*t*(20) = 2.63, *p* = 0.016, *d* = 0.57; *p*_adj_ = 0.016). These results suggest that as the SOA level increased, levels of P2 attenuation also increased.

### 3.3 Relationship Between α Exponents and N1 Enhancement/P2 Attenuation

To assess the relationship between the implicit sense of action control and its effect on sensory processing, we conducted a linear mixed effects model analysis between the *α* exponents from the MA condition and the N1 enhancement/P2 attenuation effect (see Supporting Information for full output and assumption checks). No transformations were made to the data for the purposes of meeting assumptions. For both N1 and P2 analyses, there were 63 observations and 21 groups (for 21 participants and 3 observations per participant).

For the N1 component, the model (*N1‐ C‐MA ~ 1 + α‐ MA + N1‐ AV + MA‐ IKI + (1 | Participant*)) consisted of the N1 amplitude of the corrected MA condition as the output variable, fixed effects of the *α* exponent in the MA condition, MA condition IKI, and N1 amplitude in the AV condition, and random intercepts for Participants. The main effect of *α* ‐MA was not significant, *b* = −2.02, 95% CI [−5.771, 1.725], *p* = 0.295. The random intercept variance for Participant was estimated at 2.01 (SD = 1.42) with an intraclass correlation coefficient (ICC) of 0.41. Under the specified model, these results suggest that *α* exponents in the MA condition did not have a significant effect on the degree of N1 enhancement. To more closely examine the effect of *α* exponents on the degree of N1 enhancement, we approximated the Bayes Factor using the procedure described in Wagenmakers (2007) and Masson (2011) comparing the Bayesian information criterion of two competing models. Here, the models include the original model (*N1‐ MA ~ 1 + α‐ MA + N1‐ AV + MA‐ IKI + (1 | Participant*); BIC: 290.89) as well as an alternative model that does not include the *α* exponent variable (*N1‐ MA ~ 1 + N1‐ AV + MA‐ IKI +* (*1 | Participant*); BIC: 290.99). The analysis yielded a *BF*_10_ of 1.05, meaning that there is little evidence supporting either model.

For the P2 component, the model (*P2‐ MA ~ 1 + α‐ MA + P2‐ AV + MA ‐IKI +* (*1 | Participant*)) consisted of the P2 amplitude of the corrected MA condition as the output variable, fixed effects of the *α* exponent in the MA condition, MA condition IKI, and P2 amplitude in the AV condition, and random intercepts for Participants. The main effect of *α* ‐MA was significant, *b* = −5.684, 95% CI [−9.01, −3.30], *p* = 0.002. The random intercept variance for Participant was estimated at 0.03 (SD = 0.17) with an ICC of 0.01. Under the specified model, these results suggest that *α* exponents in the MA condition significantly predicted the degree of P2 suppression.

## 4 General Discussion

In the present study, participants completed the MA, MO, and AV conditions of the self‐stimulation paradigm. In the MA condition, participants performed keypresses, each followed by a sound, across SOA levels of 0.8, 1.6, and 3.2 s per keypress. In the MO condition, they performed the same task, but the keypresses were not followed by any sounds. The ERP from the MO condition was subtracted from the ERP of the MA condition to create a motor‐controlled MA (C‐MA) ERP. In the AV condition, participants passively listened to sounds with the timing indicated by a moving visual animation. The N1 and P2 components of the AV and C‐MA condition ERPs were compared to assess the level of sensory attenuation across different SOA levels. Furthermore, we computed *α* exponents on the time series of inter‐keypress intervals to measure implicit control of keypress timing behavior. Finally, we investigated the relationship between participant *α* exponents and their level of sensory attenuation. Contrary to many attenuation studies, our results showed N1 enhancement rather than N1 suppression. The level of N1 enhancement remained similar between the SOA levels of 1.6 s and 3.2 s. On the other hand, we observed P2 suppression, replicating other self‐stimulation studies (Han et al. 2022; Horváth and Burgyán 2013), and found that the degree of P2 suppression was predicted by participant *α* exponents.

Although studies using the self‐stimulation paradigm have used a wide variety of SOAs, with the exception of SanMiguel et al. (2013), none have investigated how it may play a role in sensory attenuation, let alone justify the use for their chosen SOA level. This may partly be because we implicitly assume behavior to operate independently on the level of the trial, and for observed output variables to be the result of discrete mental states (Gilden 2001; Gomez‐Marin and Ghazanfar 2019; Huk et al. 2018). As a result, it is often assumed that the variability related to the keypressing behavior across SOAs is simply an issue to be tamed by averaging. However, from the perspective of the participant, performing keypresses with a short SOA may involve different task demands compared to a long SOA, with implications for how well participants perform at a specified SOA as well as how participants detect and adjust for deviations in their keypressing behavior. For example, the auditory system has been shown to be capable of detecting deviations from temporal regularities. Research into the mismatch negativity (MMN)—a brain response to violations of a rule that is established by sequences of sensory stimuli (Garrido et al. 2009)—shows that even during passive listening and without allocation of attention to the auditory stimuli, the auditory system is capable of detecting violations of temporal regularities (Kujala et al. 2001). However, the ability to detect irregularities in a train of stimuli may become more difficult if the SOA becomes longer. Furthermore, the fact that participants must actively produce the determined SOA level may also make it more difficult to detect temporal irregularities, especially as the required SOA becomes larger. Therefore, as SOA increases, the cognitive demands of maintaining temporal regularity for increasing SOAs may become larger. The behavioral consequences of the different SOA levels can be revealed by analyzing the variability of the keypress fluctuations across time.

Participants in the present study performed 525 keypresses for each condition and SOA level, with each string of 525 keypresses in a single block. This allowed us to analyze the fractal properties for each time series via DFA analysis (as it normally requires long time series of at least ~500 samples). Past studies on keypress and tapping behavior have shown that as the interval requirement for keypresses increases, the level of fractal behavior also increases (Madison 2004; Madison and Delignières 2009), although it should be noted that previous research has rarely used SOAs as long as three seconds. This was replicated in the present study, with *α* exponents increasing as the SOA level increased. One possible reason for this increase in persistence as SOA levels increased is that with short SOAs, deviations from the target time interval may be more easily detected by the participant, who can then make adjustments within the next keypress cycle. On the other hand, with longer SOAs, participants might only notice deviations from the target time interval after the rush or delay has accumulated over several keypress cycles. In this sense, keypress timing tendencies for longer SOAs can “persist” over time.

In our data (see Figure 3), several participants at the 3.2 SOA level yielded *α* exponents higher than 1. Theoretically, *α* exponents that exceed the value of 1 may indicate nonstationarities in the time series. However, *α* exponents that hover around the value of 1 sit within a “range of uncertainty” regarding their classification as either stationary or nonstationary (Eke et al. 2002). Therefore, some studies advise for time series to only be classified as nonstationary if they exceed the value of 1.2 due to potential biases with the DFA procedure (Arsac and Deschodt‐Arsac 2018; Delignières and Marmelat 2012; Ihlen 2012). For this reason, as none of the *α* exponents we attained exceeded 1.2, we opted to treat all time series as stationary. For the present study, exponents higher than 1 can alternatively mean that the keypress intervals in the time series are indeed of a nonstationary nature, meaning that the mean or variance changes over time. This may indicate a drop in concentration or difficulty in maintaining proper timing through the course of the trial, as the 3.2 SOA trial was of a long duration. Furthermore, when SOAs are shorter, participants may more easily keep track the timing of several keypress cycles as opposed to longer SOAs. Several studies that have investigated the relationship between IKI and fractal properties also noted nonstationarities as IKI increased, though none have used durations as long as 3.2 s (Madison 2001; Michon and Van der Valk 1967).

In addition to higher exponents as SOA increased, we also found higher exponents for the MA production condition compared to the MO condition. A lower *α* exponent (one that approaches 0.5) essentially means that the fluctuations of the keypress timings become more random, rather than a pattern of persistence or anti‐persistence (though the *α* exponents for the MO condition were still above the 0.5 level). This may partly be explained by the fact that, during the practice session, participants first listen to a series of sounds that demonstrated the required keypress SOA level. They then store this template from the practice session into memory to replicate during the actual testing session for the MA condition. However, because keypresses during the MO condition did not result in a sound, participants were not able to make use of the template during the practice session to replicate. Therefore, having the sensory feedback in memory may have allowed the participants to more easily rely on internal timing and control as they can compare their present keypress cycles on the auditory intervals from the previous cycles. The auditory system has been proven to be capable of detecting departures from temporal regularities (Garrido et al. 2009; Kujala et al. 2001). Without sensory feedback, participants may have had a more difficult time producing regular sequences of stimuli. The differences here may also serve to highlight growing concern over the use of the MO condition to serve as a control for motor processes of the MA condition (Neszmélyi and Horváth 2017; Neszmélyi et al. 2022). Neszmélyi and Horváth (2017), for example, demonstrated that participants used different amounts of force between the MA and MO conditions, thus violating the assumption of equivalent motor processes behind actions that generate sounds and actions that do not result in a sound (see also Cao et al. 2020). Here, we highlight the behavioral differences that may potentially result from the different control processes involved in generating the keypress timings. Furthermore, differences in IKI between the MA and MO conditions may also indicate potential strategies and cognitive processes in maintaining timing between the conditions. The growing concern over the uses of the MO condition also demonstrates the complex processes that go into a seemingly simple action such as a keypress. Future studies should further investigate the different dimensions of this action and how they may play a role in sensorimotor processing (e.g., Pfister et al. 2023, has suggested looking at keypress durations).

Contrary to most studies using the self‐stimulation paradigm, we found N1 enhancement rather than N1 suppression. One possible reason for this result may be the difference in temporal predictability between this study and most previous studies that have used the self‐stimulation task. Between‐condition differences in temporal predictability have been identified as a potential confound in the self‐stimulation paradigm (Hughes et al. 2013), which may explain why we saw different results here. Of the studies that attempted to control for temporal predictability, results have been mixed (Egan et al. 2023; Harrison et al. 2021; Kaiser and Schütz‐Bosbach 2018; Lange 2011; Sowman et al. 2012).

For example, a study by Lange (2011) found N1 suppression despite the fact that they used a visual cue to control for temporal predictability. Harrison et al. (2021), on the other hand, found no difference between N1 amplitudes between self‐generated sounds and sounds that were made temporally predictable via a visual cue that was similar in design to the present study. Finally, Kaiser and Schütz‐Bosbach (2018) ran an experiment wherein across all conditions (MA, MO, and the traditional *auditory‐only* condition), there was a visual countdown from 3 to 1 followed by an “X” which served as a visual cue of the event in question (such as a keypress or sound occurrence). In contrast to the previous two experiments, however, Kaiser and Schütz‐Bosbach found N1 enhancement instead. Although the reason for the mixed results is not clear, one potential explanation is the different levels of predictability achieved with the use of different methods for controlling temporal predictability. For example, Lange (2011) displayed a visual cue, in which a sound followed some time later (the delays used were 150 up to 950 ms). The present study, similar to the design used by Harrison et al. (2021), uses moving lines that cross through a fixation line to indicate the onset of a sound. However, the N1 enhancement of the present study more closely resembles the results of Kaiser and Schütz‐Bosbach's (2018) study.

Other studies that have investigated differences in how we process self‐ versus externally generated stimuli have also found mixed results. For example, in behavioral studies of sensory attenuation, participants make judgments about the loudness of sounds that are self‐generated versus passively heard sounds (Kiepe et al. 2024; Paraskevoudi and SanMiguel 2021; Reznik et al. 2015a). While Kiepe et al. (2024) found participants to judge self‐generated sounds as louder than passively heard sounds (enhancement), Reznik et al. (2015a) found the judgment to be different depending on the intensity of the self‐generated sound. When the self‐generated sound intensity was low, participants were more likely to report perceived enhancement. Conversely, participants were more likely to report perceived suppression when the self‐generated sound intensity was high. In addition, several studies that have used fMRI instead of EEG found sensory enhancement instead of sensory attenuation (Reznik et al. 2014, 2015b). The fact that we find such varied results among different paradigms investigating sensory attenuation may indicate that more investigation needs to be done into the different task demands among the different paradigms that may lead to different results (Nau et al. 2024). Furthermore, it calls into question whether “sensory attenuation” can be considered such a unitary phenomenon.

Another potential reason for the lack‐of‐N1 attenuation lies in the use of the AV condition instead of the traditional auditory‐only condition. While the moving lines helped to control temporal predictability, the neural activity represented via the ERPs may have also received contributions via the visual stimulation. Furthermore, the visual stimulation may have substantially dampened the contribution of the N1 unspecific component (Näätänen and Picton 1987; SanMiguel et al. 2013). In the study by SanMiguel et al. (2013), the authors found that the observed N1 attenuation mainly reflected the suppression of the N1 unspecific component. The N1 unspecific component has been argued to reflect cortical activity that facilitated motor activity, rather than any sensory‐specific processing. Given that self‐initiated sounds can be temporally predicted, while sounds in the traditional auditory‐only condition cannot be temporally predicted, it was argued that the suppression of the N1 unspecific component for the MA condition mainly reflected a reduced orienting response to predictable sound onset. The observed lack of N1 attenuation in the present study may reflect the fact that sounds were also temporally predictable in the AV condition, thereby eliminating the orientation response to unpredictable sounds.

Of the three SOA levels, only the 0.8 s level did not result in any differences in N1 amplitude between the C‐MA and AV conditions. The small N1 amplitudes are likely due to a phenomenon known as repetition suppression (Pereira et al. 2014; Sable et al. 2004), wherein the neural response to a stimulus decreases as a result of the stimulus being repeatedly presented at a fast pace. The dependence of the N1 amplitude on the SOA level may also reflect the phenomena of habituation or refractoriness for the N1 peak. It has been argued that the observed reduction of the N1 amplitude in repeated stimulus presentations is either the result of a learning process known as the habituation account (Rosburg and Mager 2021) or due to a recovery/refractory period for the neural generators underlying the N1 component, known as the refractoriness account (Budd et al. 1998). The N1 component amplitude has been shown to increase in amplitude as the interstimulus interval increases, likely reflecting increasing recovery of the N1 neural generators. The increased N1 in relation to increasing SOA levels may reflect this finding. Furthermore, given that participants are trying to produce regular sequences of auditory stimuli and are actively on the lookout for temporal deviations, participants may therefore become sensitive to departures from the temporal regularities they produce. The N1 response may thereby be amplified for stimulus occurrences that are tagged as departing from the regular temporal sequence, similar to an MMN response (though it has also been argued that the MMN and N1 are functionally distinct components (Campbell et al. 2007)). Because auditory stimuli are predictable in the AV condition, this could lead to the same self‐generated sound considered “deviant” not being so in the AV condition, resulting in a comparably smaller N1 response in the AV condition compared to the same self‐generated sound.

Although we observed N1 enhancement for the 1.6 and 3.2 SOA levels, the level of enhancement was not significantly different across the 1.6 and 3.2 SOA levels. Furthermore, there was no significant relationship between N1 enhancement and *α* exponents based on the linear mixed effects model analysis. Although the phenomenon of N1 suppression has long been argued to be the result of motor‐based forward models, recent literature has begun to question this interpretation (Dogge et al. 2019a; Press et al. 2023). For example, it has been argued that action effects should be distinguished between body‐generated (e.g., tickling) versus tool‐based (e.g., keypress) action effects with different predictive mechanisms distinguishing them (Dogge et al. 2019a; Press et al. 2023) Furthermore, Press et al. (2023) argued that while sensory attenuation has long been linked to our phenomenological sense of agency (Han et al. 2021), the degree of attenuation has erroneously been used as a proxy for the degree of agency. The fact that studies have demonstrated sensory enhancement when participants obviously possessed agency lends credence to this argument. Furthermore, some studies have even questioned whether N1 amplitudes are affected by motor‐based predictions (Dogge et al. 2019b; Harrison et al. 2023). That we observed N1 enhancement may help lend support to these recent studies that have argued against the phenomenon of N1 suppression being a result of forward models that “cancel out” predicted action effects (Press et al. 2020). Instead, sensorimotor interactions may involve a range of processes including both attenuation and enhancement depending on the context and likely cannot be reduced to a simple “cancellation” principle (Press et al. 2020).

We observed P2 suppression in the present study across the 1.6 and 3.2 SOA levels, though not the 0.8 level. The P2 suppression observed here replicates most studies that have investigated P2 suppression in the context of the self‐stimulation task. Furthermore, we find that as SOA increased, the degree of P2 suppression also increased, and that the degree of P2 suppression could be significantly predicted by participant *α* exponents in the MA condition. Although the N1 and P2 components have frequently been analyzed together in the self‐stimulation paradigm, compared to the N1, the functional significance of the P2 component is less clear (Crowley and Colrain 2004), with the P2 component being affected by both stimulus qualities (Steinmetzger and Rupp 2024) and higher‐order cognitive factors (Seidel et al. 2021; Sowman et al. 2012). Literature on P2 suppression has argued that it may be a marker for participant sense of agency and control (Ghio et al. 2021; Seidel et al. 2021; Timm et al. 2016). For example, Seidel et al. (2021) manipulated participants perceived levels of control of sound production by having participants complete a two‐button choice task in which they were induced to experience either high or low levels of illusion of control. Their results replicated the N1 suppression effect, although the level of suppression was not affected by the control manipulation. On the other hand, they found that P2 suppression only occurred when participants believed themselves to have high levels of control over sound production. The present study found that the degree of P2 suppression increased as SOA increased. As SOA increased, participants must enact more control over the timing of their keypresses to maintain the SOA rate, demonstrated via their increased *α* exponents. Therefore, one possibility is that the degree of P2 suppression increased as participants increased their enactment of control. However, it should be noted that the manner in which “control” is used here is different from how it is normally used in the literature, which is usually used interchangeably with sense of agency. Sense of agency is normally conceptualized within the sensory attenuation literature as causality or perhaps belief in causality regarding action effects (Haggard 2017; Seidel et al. 2021; Synofzik et al. 2008). These two aspects of sense of agency map onto the categories of *feeling of agency* and *judgment of agency* as argued by Synofzik et al. (2008). Control in the present study, however, refers to its enactment as participants cognitively engaged in the task of maintaining a control parameter (here, timing requirements of the SOA level). This implicit enactment of control, presumably involves several cognitive processes such as working memory and duration estimation. This may explain why we see effects of this “control” with the P2 component but not the N1 component. The N1 component is thought to mainly reflect acoustic changes or stimulus qualities (Näätänen and Picton 1987; Steinmetzger and Rupp 2024), while the P2 component seems to be involved with a range of processes including higher‐order cognitive processes such as working memory (Duzcu et al. 2019; Lefebvre et al. 2005).

It has been demonstrated that P2 amplitudes increase as a function of increasing SOAs (Pereira et al. 2014). When examining the P2 components across production conditions and SOAs, we can see that P2 amplitudes increase in the AV condition as a function of the SOA level. However, regardless of the SOA level, P2 amplitude remains relatively stable for the C‐MA condition. P2 amplitudes have been shown to be affected by attention, with a review by Crowley and Colrain (2004) noting that an increase in attention reduces P2 amplitudes. One possibility is that attention for sounds in the AV condition decreased due to the increasing length of the block as SOA levels increased, thereby leading to an increased P2 amplitude as the SOA increased. Conversely, for the conditions involving keypressing behavior (MA/MO conditions), participants have to generate a temporally regular sequence of events. Dynamic attending theory (Jones 1976) argues that motor activity serves to synchronize fluctuations of attention with the regular timing of events in order to enhance sensory gain (Morillon and Baillet 2017; Morillon et al. 2014). Therefore, during the production of regular sound sequences, attention toward sound onset may be enhanced in time with keypressing behavior in order to facilitate processing of the auditory stimuli. This explanation may also have a role in explaining the effects of the observed N1 enhancement, as an increase in attentiveness can increase the amplitude of the N1 component while decreasing the amplitude of the P2 component (Crowley and Colrain 2004). Furthermore, although the role is not clear, it is also possible that the long study length and large number of trials may have also affected the observed N1/P2 components. Future studies may want to systematically investigate how the N1/P2 components develop over long strings of trials (Cao, Veniero, et al. 2017b; Elijah et al. 2018).

Apart from the present study, the only other study to our knowledge that has investigated SOAs in the self‐stimulation paradigm is SanMiguel et al. (2013). In their study, they found that N1 suppression was increased with their long SOA condition (3.2 s), while they found equal levels of P2 suppression across all SOA levels. It should be noted, however, that their experimental design did not include a control for temporal predictability, which has been argued to be a potential confound in the self‐stimulation experiment (Hughes et al. 2013). Given the divergent results between past studies and more recent studies that have controlled for temporal predictability, this makes it difficult to compare the results of both SOA studies. However, it can be argued that SOAs do indeed play a role in sensory attenuation, especially because participant behavior is likely different under different contexts even if the action of performing a keypress is the same.

For example, in the self‐stimulation paradigm, there are studies where participants perform strings of keypresses at a certain tempo (like the present one) as well as studies where they perform a single keypress and not according to any prescribed SOA level (Han et al. 2021; Poonian et al. 2015). The first type of study requires that participants maintain temporal attention (Morillon et al. 2014) as opposed to the second type and so requires implicit action control. Future studies should keep in mind the different task demands between these types of studies and how different observed effects fit within the umbrella of “sensory attenuation.” Furthermore, within studies that require participants to maintain a certain SOA for keypresses, SOA requirements can vary widely within a single experiment (e.g., participants in Saupe et al. (2013) were required to perform a keypress every 1.8 s–5 s). The results of this study suggest that participants exercise different levels of control at different SOA levels and that these differences have an effect on component amplitudes. Therefore, future studies that require participants to perform keypresses at a particular rate should carefully consider the implications of their choice. However, it may also be of interest to investigate if participants have individual preferences in keypress timing tempo and whether they correspond to any differences in neural processing.

Few studies have examined how fractal properties of behavior relate to neural processes. Although the present study attempts to do so, a limitation is that we have only looked at the correlation between sensory attenuation and the fractal indices of participant keypress behavior. A follow‐up study could be one in which SOA levels are maintained but participant keypress behavior is still constrained in some way (e.g., by making participants only perform a keypress through evenly spaced moving lines like in the AV condition, thereby constraining participant enactment of control). This could also help to control for the previously mentioned MMN‐like deviations in timing that may affect ERP component amplitudes as participants are no longer concerned about producing accurately timed sequences of sounds. Perhaps another limitation is the study length: participants completed three sessions of three series of 525 keypresses per session. Although the order of SOAs and conditions was counterbalanced, the different lengths of the SOA levels may have caused attentional differences. Finally, although the widely used DFA analysis normally requires a time series of at least 500 samples, recent methods, such as the unbiased DFA (Yuan et al. 2018) have been developed that allow for shorter time series of about 300 samples. However, more testing should be done to verify the validity of the method.

It is also worth mentioning that the experimental paradigm is not typical of most studies using the self‐stimulation task. For example, rather than a single testing session, participants completed sessions across three days. One issue from this is that within‐person variability may be increased due to differences in alertness, fatigue, and enthusiasm across testing sessions. We tried to address this by making sure that participants completed sessions at the same time for each day and to make sure that there was no more than a single day between sessions. A related issue is that there may also be increased variability due to minor differences in cap and electrode placement. Therefore, topographies and differences between ERPs may be impacted by differences between electrode placements. We tried to ensure consistent cap/electrode placement by having the experimenter take photographs of cap placement for participants across testing sessions (however, this may not have completely prevented minor inconsistencies in cap/electrode placement). Finally, whereas the MA and MO conditions only involved a single vertical line, the AV condition involved participants tracking multiple moving lines. While this may help in controlling for temporal predictability, it is also different from the *passive* (or comparison) condition in previous studies. For example, there may be differences in attention between the AV condition and the *passive* condition in previous studies and may make it more difficult to compare results across studies.

In conclusion, the results of the study suggest that the N1 component may not be affected by motor‐based predictions, in line with recent studies, as we had found N1 enhancement after controlling for temporal predictability. Furthermore, we found P2 suppression, with the degree of suppression correlating with the amount of control participants exercised over their keypressing behavior as SOA increased. Whether or not this enactment of control plays a key role in P2 suppression is an open question and may be a worthwhile question for future studies.

## Author Contributions

**Nathan Thomas Han:** conceptualization, formal analysis, investigation, methodology, software, writing – original draft, writing – review and editing. **Tingting Yan:** investigation, methodology, writing – review and editing. **Ran Zhuang:** investigation, methodology, software. **Athanasios Vasileios Kokkinakis:** methodology, writing – review and editing. **Liyu Cao:** conceptualization, funding acquisition, methodology, supervision, writing – review and editing.

## Disclosure

Code availability: EEG analyses were performed using EEGLab and ERPLab. Statistical analyses were performed using JASP version 0.19.1. Scripts for keypress analyses are available from OSF (https://osf.io/7f2wr/).

## Conflicts of Interest

The authors declare no conflicts of interest.

## Supporting information


**Data S1:** Supporting Information.

## Data Availability

Raw and preprocessed data and materials are available from OSF (https://osf.io/7f2wr/).

## Endnotes

^1^

*α* exponents that lie between 1 and 2 may indicate that the time series is nonstationary, which we further explain in Section 2.4.

^2^
Since not all studies measuring the autocorrelation of behavior use the *α* exponent, we will simply use the term “fractal behavior” when discussing these studies. “More fractal behavior” is equivalent to having a higher *α* exponent.
