# Supplementary material for: Sensory Attenuation of Auditory P2 Responses is Modulated by the Sense of Action Timing Control
Source: Psychophysiology. 2025 Sep 3;62(9):e70134. doi: 10.1111/psyp.70134 (PMC12406647; doi:10.1111/psyp.70134)
Supplement: Supplementary file 1 — Data S1: Supporting Information. [file PSYP-62-e70134-s001.docx]

**Supplementary Materials**

**Detrended fluctuation analysis and α exponent explanation**


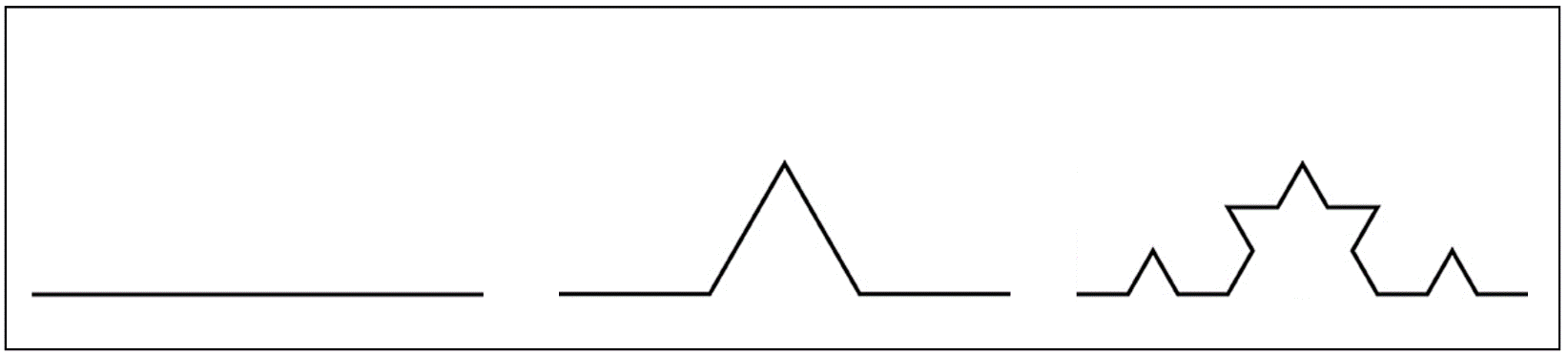
The logic for using fractal analyses is that the fluctuations of physiological time series are not completely random (Gilden, 2001). The fluctuations of physiological time series are said to result from an interaction between external influences, such as environmental constraints/task demands and actions taken by the agent attempting to maintain an equilibrium state; these fluctuations have been argued to contain fractal properties which can include *scale-invariance*, *self-similarity*, and *power law scaling relationships* (Eke et al., 2002). These properties can be captured using fractal analyses such as the DFA. The first two properties of scale-invariance and self-similarity are illustrated in the von Koch curve below (*Figure 1*).

**Figure 1***.* The von Koch curve is constructed by iterating a simple algorithm (Likens & Stergiou, 2020). From a straight line, remove the middle third and replace the segment with two sides of an equilateral triangle. This can be repeated infinitely for each straight line segment of the new construction. The von Koch curve, like all pure fractals, illustrate three related concepts known as *self-similarity*, scale*-invariance*, and *power law scaling relationships*. Self-similarity means that smaller parts of the fractal object resemble the shape formed by the whole object. Scale-invariance means that the same patterns appear regardless of the level one magnifies the structure.

The third feature of fractals, power law scaling relationships, can be illustrated with equation 1 (Eke et al., 2002):

*q = ps^ɛ^* (1)

where *q* stands for a quantitative property, *s* stands for the scale or scaling quantity, *ɛ* the scaling exponent, and *p* an initial factor of proportionality which determines the magnitude of *q*. Using the example of the von Koch curve, if quantitative property *q* is length and if *s* is a ruler used to measure length, then *q* exhibits a scaling relationship with *s* in that as *s* continuously decreases in size (so that the measurement ruler gets increasingly smaller), *q* can increase without limit as the decreasing scale allows for new infolds of the structure to be revealed. *ɛ* the scaling exponent serves to quantify the fractal elements of the system (how measured property *q* changes as the scale *s* changes). The value of *ɛ* can be determined as the slope of a linear regression line fit to data pairs on a plot of log *q* versus log *s* (Eke et al., 2002):
 log *q* = log *p* + *ɛ* log *s*  (2)

Although there exists different classes of fractal analyses, they all have in common the goal of determining the scaling exponent via plotting data pairs of log *measured property* versus log *scale* then finding the regression slope. The DFA analysis was introduced by Peng et al. (1994) to address the issue of nonstationarity (e.g., changes in mean or variance) in time series data by adding a detrending step in the analysis (step three in the DFA procedure above). Since physiological time series can sometimes be nonstationary (Eke et al., 2002), DFA is a highly popular method for fractal analyses of physiological time series as it is a method capable of dealing with both stationary and nonstationary data (Delignières & Marmelat, 2012; Likens & Stergiou, 2020).

Unlike pure fractals like the von Koch curve, natural fractal objects such as physiological time series are statistically fractal. Rather than exact self-similarity, physiological time series are *­self-affine* (although they are still commonly referred to as self-similar in the literature (Eke et al., 2002)) so that under different scales of observation, one can still see similar statistical properties such as variability and correlation structure.

Referring back to the DFA analysis as an example, the time series is divided into windows of size *n*, which is equivalent to the different scales of observation, with the RMS residual (characterising variability) calculated for each window. While pure fractals have the property of scale-invariance, so that the scale can continuously become smaller, natural fractals have limits for the size of the scale. When one plots the pairs for each window size *n* and its equivalent RMS residual into a log-log plot, the slope of the regression line fitted onto the plot reveals the fractal dimension, here the DFA exponent α. Theoretically, an α exponent > 1 may indicate nonstationarities in the time series that may be addressed with further preprocessing of the original time series (Likens & Stergiou, 2020). However, α exponents that hover around the value of 1 are said to sit within a “range of uncertainty” regarding their classification as either stationary or nonstationary due to possible biases in the procedure (Delignières et al., 2016; Eke et al., 2002). For this reason, several studies have advised for time series to only be classified as nonstationary if they exceed the value of 1.2 (Arsac & Deschodt-Arsac, 2018; Delignières & Marmelat, 2012; Ihlen, 2012).

**Linear Mixed Effects Model Outputs**

**N1**

| Model Info | | | |
| --- | --- | --- | --- |
| **Info** | |  | |
| Estimate |  | Linear mixed model fit by REML |  |
| Call |  | N1-CMA ~ 1 + N1- AV + Hurst- MA + MA IKI+( 1 \| Participant ) |  |
| AIC |  | 277.776 |  |
| BIC |  | 290.886 |  |
| LogLikel. |  | -133.014 |  |
| R-squared Marginal |  | 0.519 |  |
| R-squared Conditional |  | 0.715 |  |
| Converged |  | yes |  |
| Optimizer |  | bobyqa |  |
|  | | | |

## Model Results

| Fixed Effect Omnibus tests | | | | | | | | | |
| --- | --- | --- | --- | --- | --- | --- | --- | --- | --- |
|  | | **F** | | **Num df** | | **Den df** | | **p** | |
| N1- AV |  | 26.16 |  | 1 |  | 56.8 |  | < .001 |  |
| Hurst- MA |  | 1.12 |  | 1 |  | 54.2 |  | 0.295 |  |
| MA IKI |  | 3.10 |  | 1 |  | 54.8 |  | 0.084 |  |
| Note. Satterthwaite method for degrees of freedom | | | | | | | | | |
|  | | | | | | | | | |

| Fixed Effects Parameter Estimates | | | | | | | | | | | | | | | |
| --- | --- | --- | --- | --- | --- | --- | --- | --- | --- | --- | --- | --- | --- | --- | --- |
|  | | | | | | **95% Confidence Interval** | | | |  | | | | | |
| **Names** | | **Estimate** | | **SE** | | **Lower** | | **Upper** | | **df** | | **t** | | **p** | |
| (Intercept) |  | -3.528 |  | 0.377 |  | -4.267 |  | -2.7895 |  | 19.3 |  | -9.36 |  | < .001 |  |
| N1- AV |  | 0.797 |  | 0.156 |  | 0.491 |  | 1.1022 |  | 56.8 |  | 5.11 |  | < .001 |  |
| Hurst- MA |  | -2.023 |  | 1.912 |  | -5.771 |  | 1.7251 |  | 54.2 |  | -1.06 |  | 0.295 |  |
| MA IKI |  | -0.558 |  | 0.317 |  | -1.179 |  | 0.0629 |  | 54.8 |  | -1.76 |  | 0.084 |  |
|  | | | | | | | | | | | | | | | |

| Random Components | | | | | | | | | |
| --- | --- | --- | --- | --- | --- | --- | --- | --- | --- |
| **Groups** | | **Name** | | **SD** | | **Variance** | | **ICC** | |
| Participant |  | (Intercept) |  | 1.42 |  | 2.01 |  | 0.407 |  |
| Residual |  |  |  | 1.71 |  | 2.93 |  |  |  |
| Note. Number of Obs: 63 , groups: Participant 21 | | | | | | | | | |
|  | | | | | | | | | |

## Assumption Checks

| Test for Normality of residuals | | | | | |
| --- | --- | --- | --- | --- | --- |
| **Test** | | **Statistics** | | **p** | |
| Kolmogorov-Smirnov |  | 0.104 |  | 0.474 |  |
| Shapiro-Wilk |  | 0.932 |  | 0.002 |  |
|  | | | | | |

### Q-Q Plot


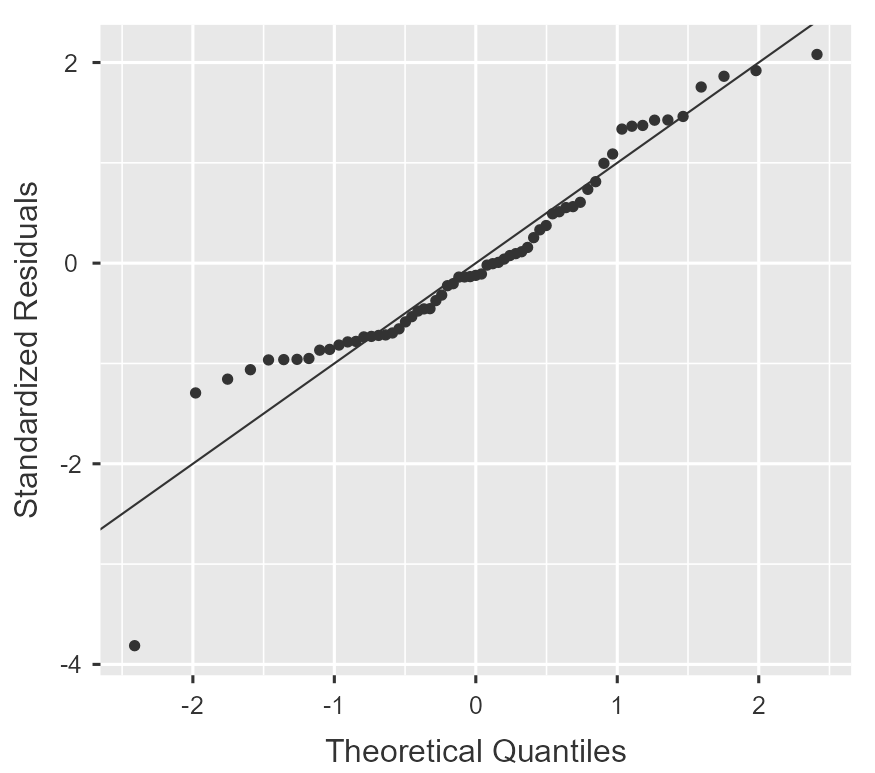


**P2**

| Model Info | | | |
| --- | --- | --- | --- |
| **Info** | |  | |
| Estimate |  | Linear mixed model fit by REML |  |
| Call |  | P2-CMA ~ 1 + Hurst- MA + MA IKI + P2- AV+( 1 \| Participant ) |  |
| AIC |  | 260.969 |  |
| BIC |  | 276.522 |  |
| LogLikel. |  | -125.832 |  |
| R-squared Marginal |  | 0.636 |  |
| R-squared Conditional |  | 0.640 |  |
| Converged |  | yes |  |
| Optimizer |  | bobyqa |  |
|  | | | |

## Model Results

| Fixed Effect Omnibus tests | | | | | | | | | |
| --- | --- | --- | --- | --- | --- | --- | --- | --- | --- |
|  | | **F** | | **Num df** | | **Den df** | | **p** | |
| Hurst- MA |  | 10.8 |  | 1 |  | 56.8 |  | 0.002 |  |
| MA IKI |  | 10.9 |  | 1 |  | 58.5 |  | 0.002 |  |
| P2- AV |  | 104.5 |  | 1 |  | 24.3 |  | < .001 |  |
| Note. Satterthwaite method for degrees of freedom | | | | | | | | | |
|  | | | | | | | | | |

| Fixed Effects Parameter Estimates | | | | | | | | | | | | | | | |
| --- | --- | --- | --- | --- | --- | --- | --- | --- | --- | --- | --- | --- | --- | --- | --- |
|  | | | | | | **95% Confidence Interval** | | | |  | | | | | |
| **Names** | | **Estimate** | | **SE** | | **Lower** | | **Upper** | | **df** | | **t** | | **p** | |
| (Intercept) |  | 3.451 |  | 0.2293 |  | 3.001 |  | 3.900 |  | 17.6 |  | 15.05 |  | < .001 |  |
| Hurst- MA |  | -5.684 |  | 1.7284 |  | -9.071 |  | -2.296 |  | 56.8 |  | -3.29 |  | 0.002 |  |
| MA IKI |  | -0.879 |  | 0.2657 |  | -1.400 |  | -0.358 |  | 58.5 |  | -3.31 |  | 0.002 |  |
| P2- AV |  | 0.838 |  | 0.0820 |  | 0.678 |  | 0.999 |  | 24.3 |  | 10.22 |  | < .001 |  |
|  | | | | | | | | | | | | | | | |

| Random Components | | | | | | | | | |
| --- | --- | --- | --- | --- | --- | --- | --- | --- | --- |
| **Groups** | | **Name** | | **SD** | | **Variance** | | **ICC** | |
| Participant |  | (Intercept) |  | 0.172 |  | 0.0296 |  | 0.00909 |  |
| Residual |  |  |  | 1.796 |  | 3.2250 |  |  |  |
| Note. Number of Obs: 63 , groups: Participant 21 | | | | | | | | | |
|  | | | | | | | | | |

##
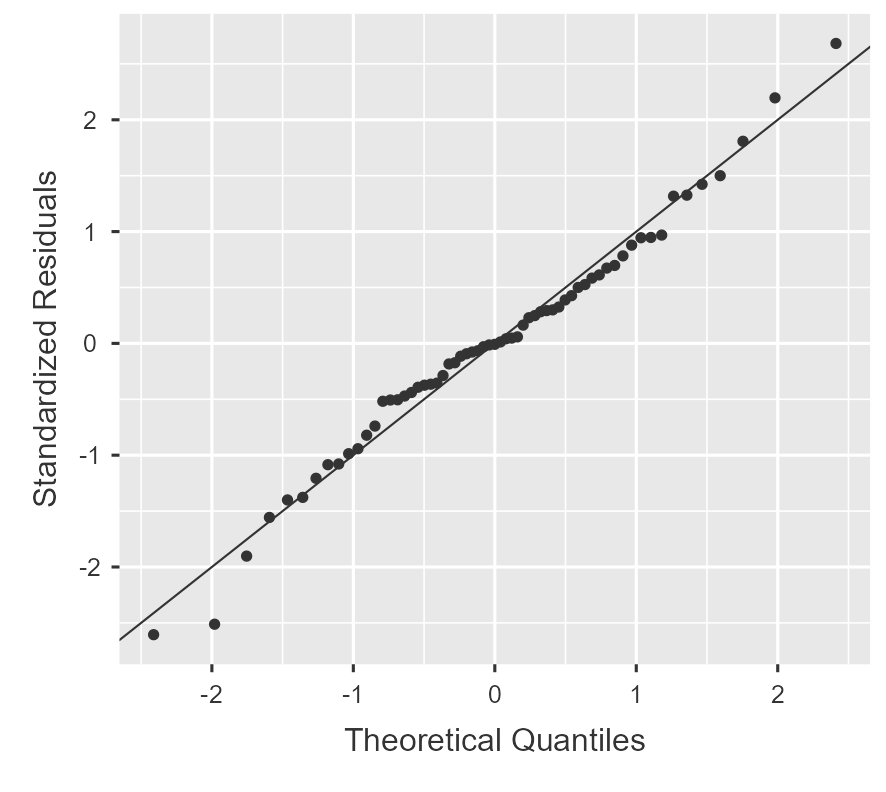
Assumption Checks

| Test for Normality of residuals | | | | | |
| --- | --- | --- | --- | --- | --- |
| **Test** | | **Statistics** | | **p** | |
| Kolmogorov-Smirnov |  | 0.0960 |  | 0.574 |  |
| Shapiro-Wilk |  | 0.9838 |  | 0.577 |  |
|  | | | | | |

Q-Q Plot

**References**

Arsac, L. M., & Deschodt-Arsac, V. (2018). Detrended fluctuation analysis in a simple spreadsheet as a tool for teaching fractal physiology. *Advances in Physiology Education*, *42*(3). <https://doi.org/10.1152/advan.00181.2017>

Delignières, D., & Marmelat, V. (2012). Fractal Fluctuations and Complexity: Current Debates and Future Challenges. *Critical Reviews in Biomedical Engineering*, *40*(6), 485–500. <https://doi.org/10.1615/CritRevBiomedEng.2013006727>

Delignières, D., Torre, K., & Lemoine, L. (2016). Methodological issues in the application of monofractal analyses in psychological and behavioral research. In *Nonlinear Dynamical Systems Analysis for the Behavioral Sciences Using Real Data* (pp. 573-596). CRC Press.

Eke, A., Herman, P., Kocsis, L., & Kozak, L. R. (2002). Fractal characterization of complexity in temporal physiological signals. *Physiological Measurement*, *23*(1), R1–R38. <https://doi.org/10.1088/0967-3334/23/1/201>

Gilden, D. L. (2001). Cognitive emissions of 1/f noise. *Psychological review*, *108*(1), 33. [10.1037/0033-295x.108.1.33](https://doi.org/10.1037/0033-295x.108.1.33)

Ihlen, E. A. F. (2012). Introduction to Multifractal Detrended Fluctuation Analysis in Matlab. *Frontiers in Physiology*, *3*. <https://doi.org/10.3389/fphys.2012.00141>

Likens, A. D., & Stergiou, N. (2020). A tutorial on fractal analysis of human movements. In *Biomechanics and Gait Analysis*, 313–344. <https://doi.org/10.1016/B978-0-12-813372-9.00010-5>

Peng, C.-K., Buldyrev, S. V., Havlin, S., Simons, M., Stanley, H. E., & Goldberger, A. L. (1994). Mosaic organization of DNA nucleotides. *Physical Review E*, *49*(2), 1685–1689. <https://doi.org/10.1103/PhysRevE.49.1685>
